# Immunotherapeutic potential of Crotoxin: anti-inflammatory and immunosuppressive properties

**DOI:** 10.1186/s40409-018-0178-3

**Published:** 2018-12-17

**Authors:** Marco Aurélio Sartim, Danilo Luccas Menaldo, Suely Vilela Sampaio

**Affiliations:** Departamento de Análises Clínicas, Toxicológicas e Bromatológicas, Faculdade de Ciências Farmacêuticas de Ribeirão Preto, Universidade de São Paulo, Ribeirão Preto-SP, 14040-903 Brazil

**Keywords:** *Crotalus durissus terrificus*, Crotoxin, Innate immunity, Adaptative immunity, Inflammation

## Abstract

For the past 80 years, Crotoxin has become one of the most investigated isolated toxins from snake venoms, partially due to its major role as the main toxic component in the venom of the South American rattlesnake *Crotalus durissus terrificus*. However, in the past decades, progressive studies have led researchers to shift their focus on Crotoxin, opening novel perspectives and applications as a therapeutic approach. Although this toxin acts on a wide variety of biological events, the modulation of immune responses is considered as one of its most relevant behaviors. Therefore, the present review describes the scientific investigations on the capacity of Crotoxin to modulate anti-inflammatory and immunosuppressive responses, and its application as a medicinal immunopharmacological approach. In addition, this review will also discuss its mechanisms, involving cellular and molecular pathways, capable of improving pathological alterations related to immune-associated disorders.

## From toxicology to medicinal immunopharmacology

*Crotalus durissus* is the main rattlesnake specie distributed in South America and is responsible for several snakebite accidents with considerable mortality rate. This is due to an intense systemic action characterized by prominent effects as neurotoxicity, coagulation disturbs and myalgic symptoms, leading to end-organs failure and death [1–3]. *Crotalus durissus* is also classified into subspecies, with variability in venom compositions that may result in differences of intensity and characteristics related to the clinical manifestations [2, 4]. Among the venom components, Crotoxin from *Crotalus durissus terrificus (*C.d.t.) presents a great interest in toxinology due to its pathophysiological and therapeutic implications in envenomation. Crotoxin was the first venom toxin purified and crystallized back in 1938 [5]. The toxin is the main component of C.d.t. venom (about 60% of its dry weight) and comprises a heterodimeric non-covalently bound complex formed by a basic enzymatically active phospholipase A_2_ (named CB) and an acidic non-enzymatic domain (named CA or Crotapotin) [6–8]. Early findings placed Crotoxin as the major toxic component of C.d.t. venom, capable of inducing a peripheral neuromuscular paralysis and cardio/respiratory failure, one of its main lethal effects [9–12]. Studies also showed that Crotapotin lacks toxicity or enzymatic activity but serves as a ‘chaperone’ by potentiating the phospholipase A_2_ activity of CB and the toxicity of the Crotoxin complex [8, 13–15]. Recent findings have demonstrated that Crotoxin-rich phenotype venoms are the main responsible for the high lethal toxicity of rattlesnake accidents [16].

Since the first studies, the abundance of Crotoxin in *Crotalus durissus terrificus* crude venom has lead researchers to investigate its association with C.d.t. venom biological effects. Therefore, reports on basic research and C.d.t. envenomation clinical manifestations have provided introductory findings that pointed out possible targets for Crotoxin study. Among them, the neuromuscular activity (characterized by blockade of the skeletal neuromuscular transmission and resulting in peripheral paralysis) and toxicological effects (such as myotoxicity, cardiotoxicity and lung dysfunction) have been widely investigated and associated with C.d.t. venom effects [17–22].

However, these associations have not only been restricted to the pathophysiological subject. Reports on clinical observations of victims of C.d.t. envenomations describe minor inflammatory signs and symptoms at the site of the bite, devoid of edema or redness. Patients also reported sensation of local paresthesia [23–25]. Another interesting finding is related to C.d.t. antivenom production. Early findings have shown that C.d.t. venom stimulates lower levels of protective antibodies compared to other snake venoms, raising the hypotheses of the presence of immunosuppressive component(s) within the venom [26, 27]. Aside from clinical data, experimental reports on basic research have also demonstrated that C.d.t. venom is responsible for inducing anti-inflammatory, immunosuppressive and analgesic effects [28–32]. Therefore, since the late 1980’s, toxinologists in the immunology field have been focusing in Crotoxin’s participation in both innate and adaptative immunity.

**Fig. 1:**
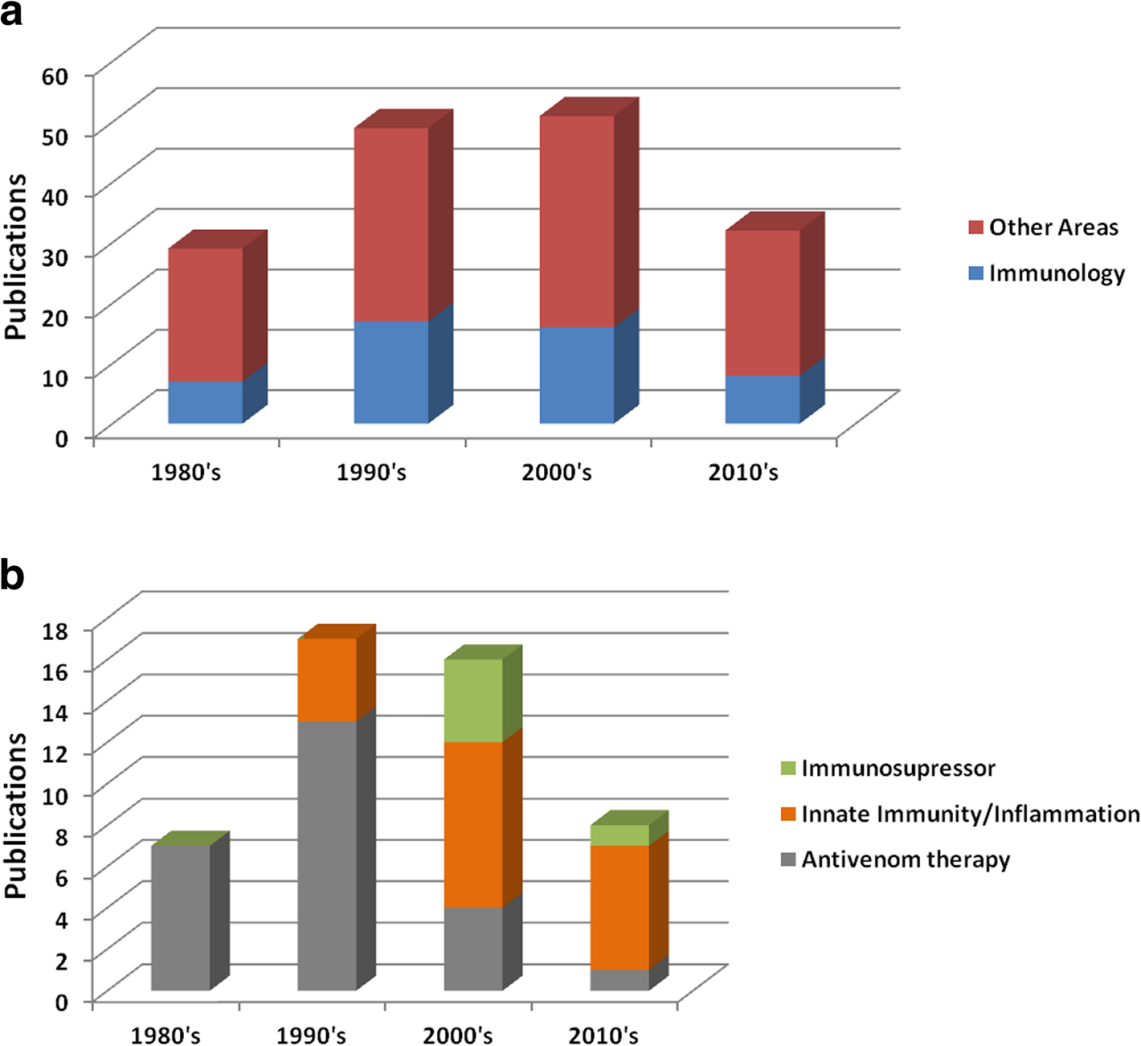
Scientific researches on Crotoxin over the years. Bibliographical survey on Pubmed (https://www.ncbi.nlm.nih.gov/pubmed/) using the keyword “Crotoxin” performed on July/2018, considering articles since 1980 and approaching biological effects associated with Crotoxin as the main focus. Initially, a total of 161 articles were categorized in immunological (48 articles) or other areas (113 articles) (**a**). Then, the 48 articles in the immunological category were divided in innate immunity/inflammation, immunosuppressive or antivenom therapy (**b**)

In order to understand the research progression on the immunologic effects of Crotoxin over the years, a bibliographical survey was performed on PubMed (searching for “Crotoxin”) to identify articles with Crotoxin as main subject in different areas (Fig. 1). Figure 1a shows an increasing number of researches involving the participation of Crotoxin in the immunology field since the 1980’s. During the 1980’s and 1990’s, the immune-related studies approaching antivenom therapy represented the main effort of researchers (Fig. 1b). These studies have shown that the Crotoxin complex and also the isolated CB are antigenic compounds capable of stimulating the production of antibodies that neutralize the lethal potency of both Crotoxin and *Crotalus durissus terrificus* venom [33–35]. These findings justify the use of the phospholipase A_2_ CB, with low toxicity compared to Crotoxin, instead of C.d.t. crude venom, as the antigen for anti-*Crotalus* antivenom production [33, 36]. However, from 2000 and forward, the investigations concerning the immunomodulatory effects of Crotoxin represented the main focus of toxinologists (Fig. 1b), and this shift can be associated with novel perspectives for the application of Crotoxin.

The 1990’s are considered a turning point on the researches involving Crotoxin, with researchers aiming at the applicability of Crotoxin as a possible pharmacological strategy. Thus, some studies have demonstrated the Crotoxin ability to improve pathological conditions not only related to immune-associated disturbs, but to other circumstances as well, including cancer and microbial infections [37–42]. However, the anti-inflammatory and, immunosuppressive effects of Crotoxin contemplate an important part in the efforts to study its therapeutic potential. Therefore, the present review describes the scientific investigations on the ability of Crotoxin to modulate immune responses, and its possible application as a medicinal immunopharmacological approach. In the following sections, we will discuss Crotoxin’s proposed mechanisms (involving cellular and molecular pathways) capable of improving pathological alterations associated with immunopathologies.

## Crotoxin’s anti-inflammatory properties

### From crude venom to Crotoxin

The inflammatory response to snake envenomation is considered a relevant issue for most venomous snakes, such as those from the Viperidae family, and is mostly associated with local tissue damage [43]. However, the clinical observation and experimental studies on C.d.t. venom report a low-moderate local acute inflammatory reaction mostly associated with secondary effects such as myonecrosis and organ damage rather than a direct effect of C.d.t. crude venom on the immune response [44–46]. Barraviera et al. for the first time in 1995 described the increase of the cytokine levels, especially IL-6 and IL-8, in patients bitten by *Crotalus durissus terrificus* [47]. A study by Cruz and colleagues [48] showed that mice injected with C.d.t. venom were capable to produce both pro-inflammatory (TNF-α and IL-6) and anti-inflammatory (IL-10 and IL-4) cytokines, with a lasting deviation of balance to anti-inflammatory dominance. Therefore, the neglected inflammatory response and possible installation of an anti-inflammatory environment led researchers to investigate the immunomodulatory behavior of C.d.t. venom facing inflammation and associated conditions.

Studies have shown that C.d.t. venom is able to reduce both carrageenan, bacillus Calmette-Guérin (BCG) and thioglycolate-induced mice paw edema and polymorphonuclear leukocyte infiltration [28, 32, 49]. Interestingly, C.d.t. venom presented a long-lasting “protective effect” capable of inhibiting inflammatory alterations when the venom was administrated 21 days before carrageenan, as well as a “treatment effect” when the venom was administrated 1 h after carrageenan stimulus [49]. Moreover, the studies have shown that C.d.t. immunomodulatory effect is associated with the production of anti-inflammatory mediators from the lipoxygenase pathway and activation of formyl peptide receptors [32]. Aside from a direct anti-inflammatory activity, reports have also evidenced that the venom was able to modulate macrophages functions. C.d.t. venom induced a significant inhibition of spreading and phagocytic activity of peritoneal macrophages both in vitro and in vivo [28, 50]. This suppressant behavior also presented a long-lasting effect with macrophages treated with venom 16 days before spreading and phagocytosis stimulation [28].

Crotoxin, as the main component in C.d.t. venom, have always been in the spotlight of researchers as the possible central character correlated with the crude venom biological effects. Therefore, several studies have been performed in order to evaluate Crotoxin’s contributions in anti-inflammatory response associated with C.d.t. venom. In that manner, most studies were conducted using C.d.t. crude venom and venom fractions consisting of isolated Crotoxin or fractions devoid of Crotoxin. Initially, the doses used for Crotoxin were based on the dose of C.d.t. venom with anti-inflammatory properties (~ 75 μg/Kg s.c.) [28, 49], which presented no evidence of envenomation clinical signs such as neurotoxic locomotor disability and respiratory paralysis. Considering that Crotoxin represents about 60% of C.d.t. venom dry weight, authors have explored its effect using doses varying from 35 to 45 μg/Kg s.c. [32, 51, 52]. Studies have shown that only C.d.t. venom and Crotoxin, but not other fractions, were capable of reducing paw edema and peritoneal leukocyte infiltration in carrageenan and BCG-treated mice [32, 51]. Similar findings were observed for macrophage and neutrophil function, where Crotoxin and C.d.t. venom were able to inhibit both spreading and phagocytosis activity [53, 54]. Others studies also confirmed that C.d.t. venom and Crotoxin shared the same mechanism concerning the changing in macrophage morphology and its association with lipoxigenase-derived eicosanoids [55, 56].

### Crotoxin and its subunits: Inflammation models in vivo and in vitro

In order to evaluate the role of Crotoxin and its isolated subunits CB and Crotapotin as anti-inflammatory agents, authors have used classical inflammatory models, both in vivo and in vitro. The models varied from inflammation induced by isolated agents such as lipopolysaccharide (LPS), carrageenan, chemical agents 2,4,6-trinitrobenzenesulfonic acid (TNBS), or attenuated organisms as bacillus Calmette-Guérin. The in vivo experimentation consisted on evaluating edema formation, leukocyte infiltration and phenotyping, inflammatory mediators quantification, leukocyte-endothelium interaction and tissue regeneration in different body regions such as paw, peritoneal cavity, cremaster muscle microcirculation and colon [32, 51, 52, 57]. For the in vitro model, authors have used murine bone marrow derived dendritic cell (BM-DC) culture and evaluated the capacity of Crotoxin to modulate its maturation and functional activity during LPS stimulation [57].

Murine models of inflammation are considered an essential approach for screening drugs and novel molecules with therapeutic potential. The option for the model usually relies on the type of inflammatory response to be studied and its mechanisms, in which the inflammatory stimulus have the pivotal role in this response. Some models intend to mimic human pathologic conditions or diseases, and others are used for general inflammation [58]. Nunes and colleagues [51] have used the carrageenan-induced paw edema, peritoneal leukocyte migration and leukocyte-endothelium interaction in cremaster muscle microcirculation in order to investigate Crotoxin’s anti-inflammatory response. The toxin was able to reduce paw edema and polymorphonuclear migration to peritoneum induced by carrageenan. Moreover, the toxin increased cell rolling and decreased cell adhesion to the endothelium surface cremaster muscle microcirculation. The toxin also exerts a long-lasting effect capable of reducing carrageenan acute inflammatory alterations up to 21 days after the toxin administration. Crotoxin was also able to treat carrageenan installed inflammation when administrated after the inflammatory stimulus. In addition, the pharmacological drugs Boc2 (a selective antagonist of formyl peptide receptor) and NDGA (nordihydroguaiaretic acid – an arachidonic acid 5-lipoxygenase inhibitor) were reported to revert the decrease in cell migration and leukocyte-endothelium interaction induced by the toxin [51]. This last finding associates the anti-inflammatory effect of Crotoxin with the production of the lipoxygenase-derived eicosanoid Lipoxin A_4_ (LXA_4_), with anti-inflammatory and “pro-resolving” properties. The LXA_4_ function will be further discussed.

Almeida and colleagues [52] have investigated Crotoxin’s anti-inflammatory effect on a more complex model using the acute colitis induced by TNBS, associated with inflammatory bowel disease. Crotoxin was able to down-modulate the development of colon mucosal inflammation by stabilizing the weight loss, increase in clinical scores and necrotic areas induced by TNBS. Also, Crotoxin treatment resulted in lower histological signs of inflammation (observed by recovery of epithelial and goblet cells and reduced crypt lesions and transmural inflammatory cell infiltration) as well as reduced mieloperoxidase activity in the tissue associated with the presence of infiltrated neutrophils. Crotoxin reduced the number of group 3 innate lymphoid cells (ILC3) and Th17 population, involved in the colonic inflammation and increased protective CD4^+^ Tbet^+^ T cell population. The toxin also induced a significant reduction of the inflammatory cytokines TNF-α, IL-1β and IL-6 in colonic tissue. Authors also found that TNBS-treated colonic tissue from animals treated with Crotoxin presented an increased quantification of anti-inflammatory associated mediators such as IL-10, TGF-β, LXA_4_ and PGE_2_ [52].

Crotapotin, the acid subunit of the Crotoxin complex, is known for the lack of enzymatic and neurotoxic activity and act as a chaperone potentiating the phospholipase A_2_ enzymatic activity of CB [15, 59]. Considering that endogenous secreted phospholipases A_2_ (sPLA_2_) are associated with inflammatory-related diseases [60], researchers have also focused their studies on Crotapotin as a possible anti-inflammatory agent. Landucci and colleagues [61] showed that the intraperitoneal and oral administration of Crotapotin, as well as its contralateral paw injection and co-injection with carrageenan, were able to reduce carrageenan-induced paw edema in rats. Crotapotin preserved its anti-edematogenic activity on adrenalectomized rats, indicating that its activity is independent of the release of endogenous corticosteroids. The toxin also inhibited carrageenan-induced edema in serotonin-depleted rats, ruling out the possibility of acting as a 5-HT receptor antagonist. Based on these findings, Landucci and colleagues [62] conducted a follow up study on Crotapotin’s ability to inhibit paw edema induced by sPLA_2_ from animal venoms. The authors reported that Crotapotin inhibited paw edema induced by *Naja naja* and *Apis mellifera* venom PLA_2_s, but potentiated the edematogenic effects of *Naja mocambique mocambique* venom PLA_2_. In addition, no effects were observed on *Crotalus adamanteus* venom PLA_2_. When the ability of Crotapotin to interfere in the PLA_2_ enzymatic function was evaluated, the toxin was capable of reducing *Apis mellifera* (edema inhibited by Crotapotin), *Naja mocambique mocambique* (edema potentiated by Crotapotin) and *Crotalus adamanteus* (not affected by Crotapotin) venom PLA_2_s. These findings indicate that, despite the homology between venom sPLA_2_s, Crotapotin interferes differently on each sPLA_2_, inhibiting or potentiating their edematogenic activity. Moreover, its ability to inhibit their enzymatic activity clearly indicates that the mechanisms of specific venom PLA_2_s might not be associated with their PLA_2_ activity [62].

The use of cellular models of inflammation allows the screening of a wide variety of drugs able to modulate inflammatory responses. Also, it enables researchers to deepen the studies on cellular and molecular levels, better understanding the mechanisms of action of foreign molecules [63]. Freitas and colleagues [57] performed an in vitro experiment with bone marrow dendritic cells (BM-DCs), considered important components of innate and adaptative immunity, to investigate the capacity of Crotoxin and its subunits (Crotapotin and CB) in modulating the maturation and functional activity of these leukocytes. The authors used LPS, a pathogen-associated molecular pattern (PAMP), as an agonist for triggering intracellular pathways responsible for maturation and induction of T cell priming. Regarding the inflammatory aspect of BM-DCs maturation, Crotoxin and CB subunit, but not Crotapotin, impaired the expression of MHC-II and costimulatory molecules CD40, CD80 and CD86 on BM-DCs treated with LPS. Moreover, Crotoxin and CB inhibited phosphorylation and downmodulated the activation of ERK1/2, p38-MAPKs and p65-NF-κB, involved in the upregulation of inflammatory cytokines, on LPS-treated BM-DCs. In this same manner, the authors also observed that both Crotoxin and CB were capable of reducing secretion of the pro-inflammatory cytokines IL-12, TNF-α and IL-6, as well as increasing production of IL-10, PGE_2_ and LXA_4_ (associated with an anti-inflammatory pattern) by LPS-treated BM-DCs. Moreover, the pretreatment of LPS-treated BM-DCs with Boc2 repressed all the modulation induced by Crotoxin and CB concerning expression of MHC-II and costimulatory molecules and the secretion of cytokines and eicosanoids [57].

### Modulation of leukocyte function

An important approach followed by researchers in order to better understand the immunomodulatory character of molecules consists in investigating leukocyte activities. Primal functions such as leukocyte recruitment, phagocytosis and egress from the inflamed site to lymphoid tissues are modulated by the environmental balance between inflammatory and anti-inflammatory stimulus [64]. Therefore, authors have engaged their efforts using the strategy of studying isolated leukocyte functions to better understand Crotoxin’s anti-inflammatory behavior.

As previously mentioned, C.d.t. venom reduced both the spreading and phagocytosis of macrophages and neutrophils, with Crotoxin being established as the main responsible for this behavior. Studies have shown that macrophages from immunosuppressed patients exhibit a reduction in the phagocytic uptake of apoptotic cells [65] and also that anti-inflammatory drugs reduce bacterial phagocytosis and killing by neutrophils [66], supporting that Crotoxin as well as other anti-inflammatory and immunosupressive drugs can alter leukocyte function. Reports have shown that Crotoxin and CB, but not Crotapotin, are capable of reducing the spreading and phagocitosys of macrophages facing different phagocytic stimuli (opsonized zymosan, opsonized sheep erythrocyte and non-opsonized *Candida albicans*) ex vivo and in vitro [53, 67]. Moreover, the inhibition of phagocytosis by Crotoxin and CB was impaired by the 5-lipoxygenase inhibitor Zileuton but not by indomethacin. Also, both Crotoxin and CB, but not Crotapotin, induced production of PGE_2_ and LXA_4_ but not of LTB_4_. Therefore, authors suggested that this inhibitory action is not associated with the type of receptor involved in the phagocytosis process (“Fc” for opsonized sheep erythrocytes, “C3b” for opsonized zymosan or “mannose” for non-opsonized particles of *C. albicans*), but mediated by eicosanoids derived from the activity of 5-lipoxygenase, such as LXA_4_ [55]. This effect involves cytoskeleton rearrangement in macrophages induced by F-actin reorganization and inhibition of tyrosine phosphorylation associated with a decrease in membrane-associated small Rho GTPases (RhoA and Rac1) [56].

Investigations have also been performed on bone marrow-derived neutrophils (BMN), which showed similar findings as those observed for macrophage phagocytosis. Crotoxin reduced BMN phagocytosis both in vitro and ex vivo and this effect lasted for 14 days after the toxin administration in rats. The authors also reported a decrease in tyrosine phosphorylation and polymerization of F-actin in neutrophils during the phagocytosis of opsonized zymosan particles [54].

**Fig. 2:**
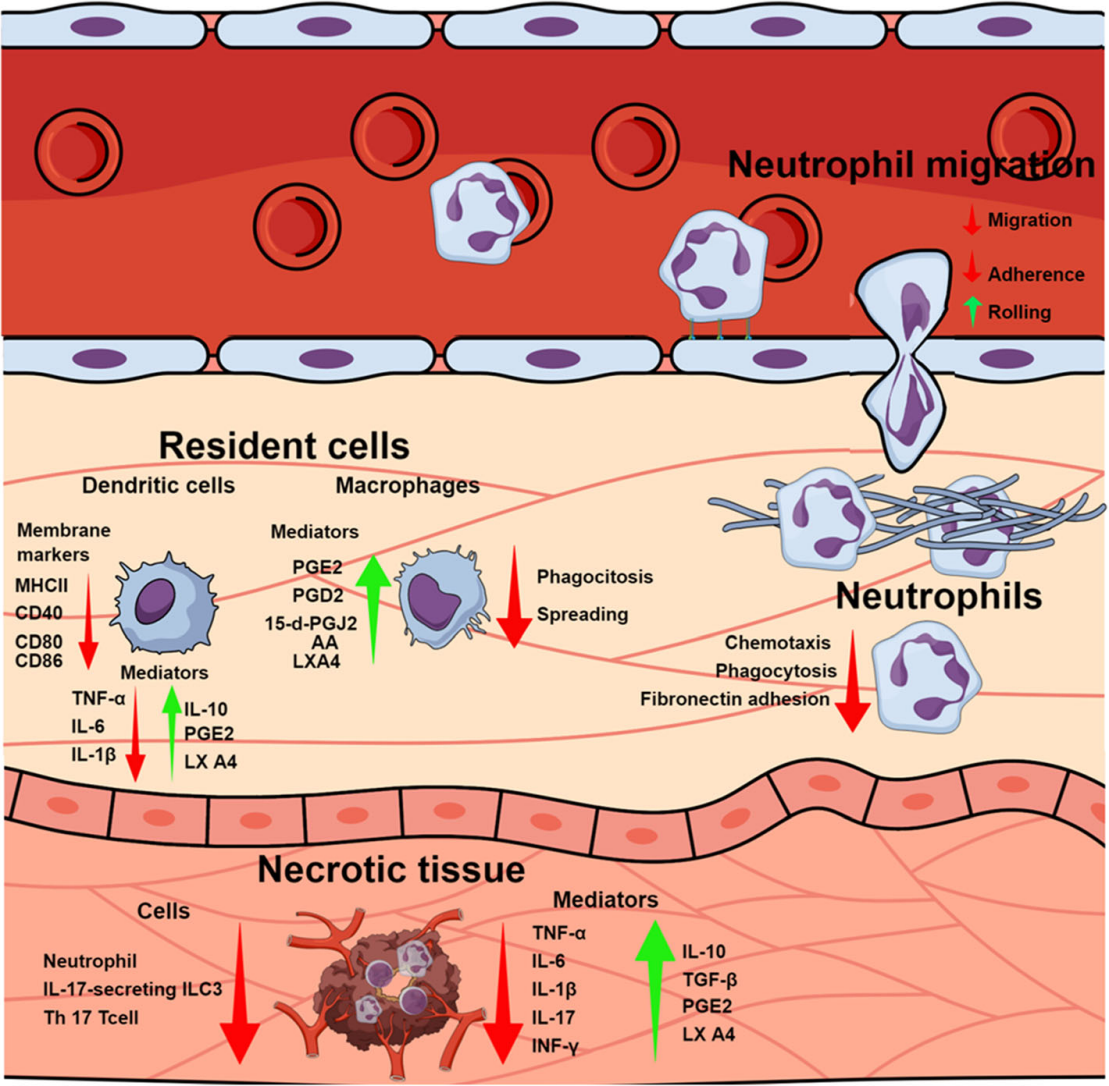
Crotoxin’s anti-inflammatory modulation. Schematic figure on circulation, tissue and cellular events modulated by Crotoxin and its subunits during inflammatory stimulus. The figure illustrates the migration (adherence, rolling and chemotaxis) and function (phagocytosis and spreading) of blood-derived, resident and tissue necrotic leukocytes, as well as the expression of pro-inflammatory (IL-1β, IL-6, IL-17, TNF-α and INF-γ), anti-inflammatory mediators (IL-10, TGF-β, PGE_2_, PGD_2_, 15-d-PGJ_2_, LXA_4_ and arachidonic acid – AA) and surface inflammatory markers (MHC-II, CD40, CD80 and CD86). Green arrows indicate events/mediators that are stimulated/increased by Crotoxin, while red arrows indicate those that are inhibited/reduced. The illustration was created using Mind the Graph platform (www.mindthegraph.com)

Lima and colleagues [68] also evaluated Crotoxin modulation on other functionalities of primed BMN. Authors showed that the toxin strongly down-regulated chemotaxis, fibronectin adhesion and phagocytosis of neutrophils stimulated with fMLP(N-formyl-Met-Leu-Phe) in vitro and ex vivo. The inhibitory character of BMN for phagocytosis lasted for 14 days after Crotoxin administration in mice. Authors also evaluated functions associated with the killing of pathogens, with Crotoxin not affecting elastase and gelatinase activity or reactive oxygen species (ROS) production by BMN, suggesting that their killing efficiency is not affected by Crotoxin although it reduces phagocytosis. Crotoxin induced down-regulation of CD11b and CD18 expression in fMLP-stimulated cells, with both membrane surface molecular markers being associated with the adhesion of neutrophils to matrix proteins and phagocytosis of opsonized particles. The same authors also evaluated the modulation of Crotoxin on signaling pathways involved in actin polymerization associated with the Syk-GTPase pathway both in vitro and ex vivo, showing that the toxin reduced the phosphorylation of signaling proteins Syk and Vav1 in fMLP-primed BMN, which are important molecules involved in the adhesion and migration of neutrophils and production of pro-inflammatory cytokines. Also, Crotoxin inhibited Cdc42, Rac1 and RhoA translocation, impairing the polymerization of G-actin into F-actin. Figure 2 summarizes the activities discussed above.

## Immunosuppressive properties of Crotoxin

The crotalic antivenom therapy consists in the production of antiserum containing protective antibodies against venom components of *Crotalus* venom. Venom immunogenicity, comprised by the capacity of inducing the production of antibodies, is fundamental for obtaining an antiserum with high neutralizing potency. Early findings have shown that although C.d.t. venom is immunogenic, it stimulates lower levels of protective antibodies compared to others snake venoms [26]. The hypothesis that the venom is composed of molecule(s) with immunosuppressive behavior encouraged researchers to investigate the participation of Crotoxin and its subunits on the humoral immune response.

Schaeffer and colleagues [27] have found that Crotalidae polyvalent antivenom presented low recognition efficiency to C.d.t. fractions with medium and low molecular weights (13–30 and < 14 k kDa, respectively). Considering that Crotoxin is present in medium and low molecular weight fractions, authors have investigated the modulation of humoral response by Crotoxin and its subunits. Cardoso and Mota [29] showed that C.d.t. venom and Crotoxin, but not CB or Crotapotin, were able to induce a significant decrease in the level of anti-chicken ovalbumin (anti-OVA) and anti-human serum albumin (anti-HSA) IgG antibodies, predominantly anti-HSA IgG1 isotype. However, the CA and CB subunits of Crotoxin did not change the antibody level to either antigen, suggesting that the suppressive effect is associated with the complex but not with the isolated subunits. Rangel-Santos and colleagues [69] demonstrated that spleen cells both T- and B-Lymphocytes from animals treated with C.d.t. venom were able to suppress IgG1 anti-HSA antibodies when injected in mice immunized with HAS, showing that the venom was also capable of modulating the cellular-axis of humoral immunity. Therefore, several other important investigations have been performed in order to better understand the capacity of Crotoxin and its subunits to modulate both cellular and humoral immunity.

As observed in previous reports described above, Favoretto and colleagues [70] have found that Crotoxin was able to suppress specific anti-HSA IgG1 and IgG2a antibody production from HAS-immunized mice, indicating its capacity to modulate Th1 and Th2 responses. In that manner, the authors observed that Crotoxin administration in HSA-immunized mice was able to reduce T cell proliferation of lymphocytes collected from inguinal and periaortic lymph nodes of these mice and re-stimulated in vitro with HSA, Concanavalin A (ConA) or anti-CD3 antibody. Other reports align with these findings, in which Rangel-Santos and colleagues [69] also observed that Crotoxin, but not CB, inhibited the cellular proliferation induced by ConcA in vitro. These authors also found that Crotoxin in vitro suppressed IL-2, IL-4, IL-10, and IFN-γ production by T cells induced by Conc-A, as well as the IL-4 and IL-10 down-regulation when the toxin was administrated in vivo. Moreover, animals treated with Crotoxin were able to suppress spleen T cell proliferation and IL-4 production from animals immunized with HSA and re-stimulation in vitro with the same antigen. Considering the modulation of Crotoxin in the cellular events of humoral immunity, Zambelli and colleagues [71] have investigated the response of lymphocytes to Crotoxin and CB treatment. The authors found that Crotoxin and CB induced lymphocyte homing to lymph nodes. Both toxins reduced the number of blood and lymph circulating lymphocytes and increased the number of T (CD3^+^) and B-lymphocytes (CD45R^+^) in mesenteric lymph node. Also, an increase in leukocyte adhesion and migration (diapedesis) in high endothelial venules (HEVs) of mesenteric lymph nodes was observed. The authors found that adhesion was mediated by L- and P-selectin, with up-regulation of lymphocyte function being associated with antigen 1 (LFA-1 - CD11a/CD18 complex), and cell adhesion with membrane surface β_2_-integrin marker. The authors also associated this lymphocyte homing effect with lipoxygenase-induced mediators, since Zileuton, a 5-lipoxygenase inhibitor, abolished the decrease in the number of circulating leukocytes and the increase in the number of leukocytes adhered to endothelial cells induced by Crotoxin and CB [71]. Another interesting finding was reported concerning another aspect of cellular events of the adaptative immunity modulated by Crotoxin. As previously discussed in the present review, Freitas and colleagues [57] showed that Crotoxin was capable of suppressing the maturation of BM-DCs. Considering dendritic cells as a crucial link between innate and adaptive immunities, authors also found that Crotoxin quenched the T cell (TCD3+) proliferation and IL-2 production induced by LPS-treated BM-DCs. This result clearly indicates the capacity of Crotoxin to modulate the antigen-presenting function of tissue resident antigen-presenting cells (such as dendritic cells), thus interfering in the adaptative immunity.

**Fig. 3:**
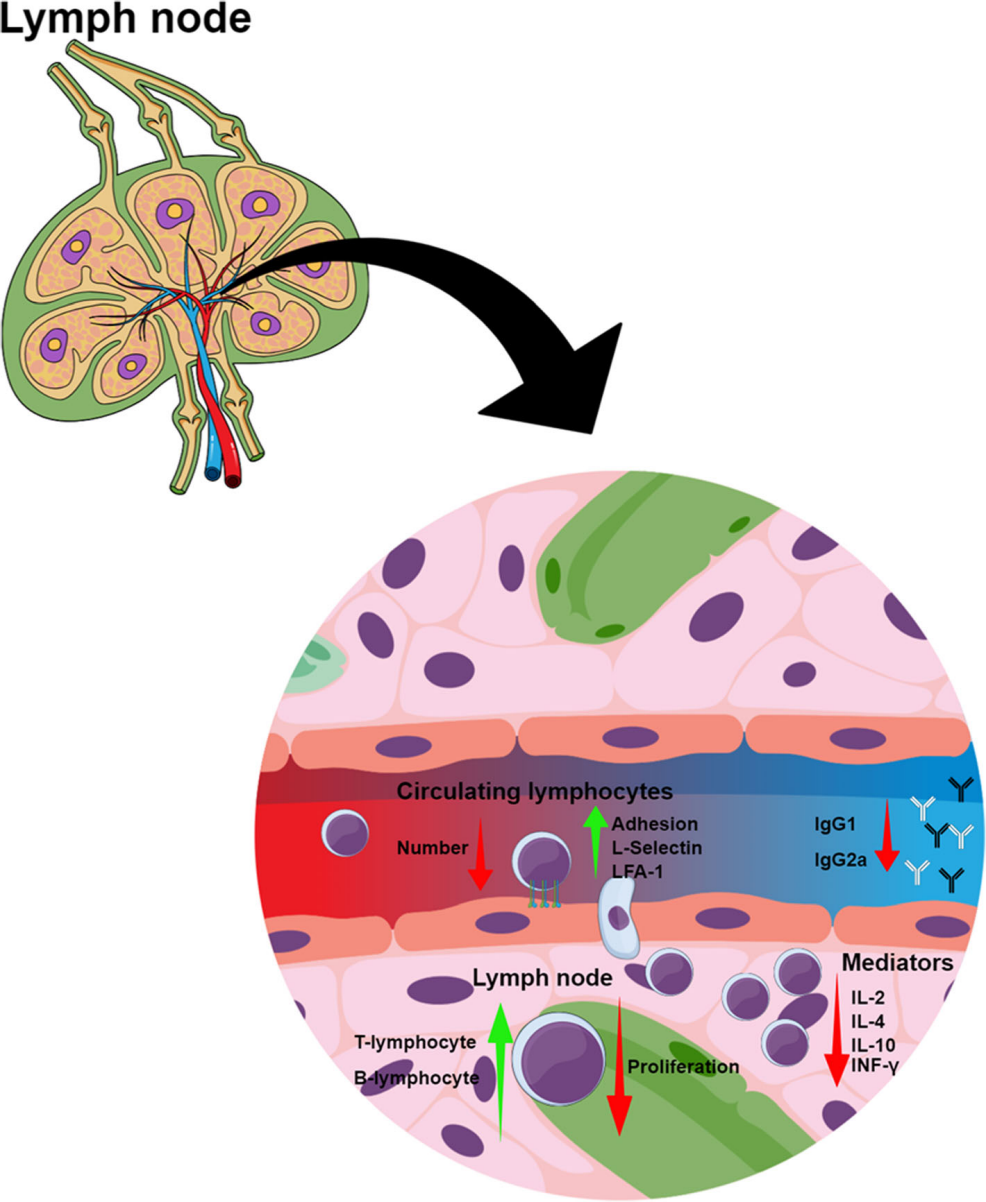
Crotoxin’s immunosuppressive modulation. Blood circulation, lymph node homing of lymphocytes and cellular events associated with the immunosuppressive effects of Crotoxin and its subunits. The figure illustrates the capacity of Crotoxin to modulate migration (adherence and chemotaxis), function (proliferation), expression of adhesion molecules (L-selectin and LFA-1), and inflammatory mediators (IL-2, IL-4, IL-10 and INF-γ) of circulating and lymph node lymphocytes, as well as imunoglobulins production (IgG1 and IgG2a). Green arrows indicate cells/events that are stimulated by Crotoxin, while red arrows indicate reduction or inhibition. The illustration was created using Mind the Graph platform (www.mindthegraph.com)

Although it was previously reported that Crotapotin presented no immunosuppressive effects [29], others studies have found different results. The experimental autoimmune neuritis (EAN) is an autoimmune T-cell-mediated neuropathy model associated with an inflammatory demyelinating disorder on peripheral nervous system that leads to paralysis [72]. Considering the anti-inflammatory and immunosuppressive properties described for the toxin, as previously reviewed [61, 62, 73], Castro and colleagues [74] evaluated Crotapotin modulation of EAN from mice immunized with P2, a peptide from peripheral nerve myelin fractions. Crotapotin was able not only to reduce clinical scores associated with the disease, but also delayed the initiation of the early effects of the pathology. This toxin was capable of reducing the infiltration of inflammatory cells into sciatic nerve. These findings indicate the effects of Crotapotin in the innate arm of the immune response associated with the EAN. Moreover, when lymph node isolated T cells from EAN-induced animals treated with Crotapotin were cultured and re-stimulated with Concanavalin A or P2 peptide, the lymphocytes presented a suppressed capacity of proliferation induced by Crotapotin. This clearly suggests a modulatory effect of Crotapotin on cellular immune response associated with regulatory T cell activation [74]. Moreover, Garcia and colleagues [73] showed that Crotapotin inhibited spleen T cell proliferation and was inhibited by indomethacin. Also, the toxin induced T cell biosynthesis of PGE_2_, associating its immnunosuppressive effect with the eicosanoid production. Figure 3 summarizes the activities discussed above.

## Crotoxin’s immunomodulatory mechanisms: the key role of pro-resolving lipid mediators

Researchers have demonstrated that the phospholipase A_2_ enzymatic activity of Crotoxin plays a key role in biological events triggered by the toxin. Toxinologists have reported that the wide range of pharmacological actions of Crotoxin is due to the hydrolysis of membrane phospholipids at critical sites, resulting in the production of biological active lipids [75–78]. The major mechanism associated with this behavior seems to involve the production of arachidonic acid-derived eicosanoids, which are lipid mediators implicated in a wide variety of biological events, such as inflammation. Each lipid mediator presents a distinct mechanism of action, being able to modulate the inflammatory response in individual manners. These unique properties are mainly associated with their capacity to act on different receptors. In addition, their metabolism and biosynthesis are regulated by environmental signals induced by endogenous or exogenous components [79].

Since the early findings, authors have associated Crotapotin’s functions to its ability to modulate CB enzymatic activity [80, 81]. Considering this and the fact that Crotapotin exhibits no toxic effects, researchers have investigated its phospholipase modulatory effects using immunological models in which endogenous or exogenous PLA_2_s play important roles. Thus, authors have found that Crotapotin was able to inhibit paw edema induced by animal venom PLA_2_s, in a catalytic-dependent manner [62, 82]. Moreover, this toxin also inhibited carrageenan-induced paw edema in rats, independently of corticosteroid production or serotonin inhibition, which was associated to a possible modulation of prostaglandin synthesis by cyclooxygenase (COX) enzymes [61]. Garcia and colleagues [73] have shown that Crotapotin induced PGE_2_ production by macrophages and lymphocytes, and associated this event with the inhibition of T cell proliferation. Considering that PGE_2_ is a well-known eicosanoid that presents a paradoxical status as a pro-inflammatory agent with immunosuppressive activity [83], it’s feasible to estimate that Crotapotin could act on PGE_2_ biosynthesis, triggering the effects caused by this lipid mediator. Nevertheless, Crotapotin’s immunomodulatory effects and mechanisms should be further investigated in order to allow clearer conclusions.

As for Crotoxin and CB, their intrinsic capacity of generating arachidonic acid by the phospholipase enzymatic activity on cell membrane phospholipids, also inducing the expression of eicosanoids, is reported as the main immunomodulatory mechanism of both molecules. Considering CB as the responsible for the catalytic activity of Crotoxin, studies using this subunit have been performed in order to understand the biosynthesis of lipid mediators by leukocytes. Moreira and colleagues [84] reported that CB was able to induce both PGE_2_ and PGD_2_ synthesis when administrated in mice peritoneal cavity, without modulating COX-1 and -2 expression in peritoneal leukocytes. The same pattern of COX expression was also observed for CB-treated macrophages in vitro, however, the toxin increased the COX-1 activity. Also, CB induced culture macrophages to produce arachidonic acid (AA), PGE_2_ and PGD_2_, in which endogenous Ca^2+^-independent PLA_2_s (iPLA2s) were found to mediate both AA and PGD_2_ biosynthesis. These results suggest that AA released by CB is supplied to COX-1 for the production of PGE_2_ and PGD_2_ [85]. Giannotti and colleagues [85] have then deepened the investigations on the production mechanisms of lipid mediators. These authors showed that CB induced peritoneal thioglycolate-elicited culture macrophages to produce lipid droplets (LDs), lipid-rich organelles that compartmentalize key enzymes involved in the metabolism and synthesis of lipid mediators. CB induces expression of PLIN2, an important structural protein associated with the formation of LDs, and its recruitment into LD cytosol. The CB-induced LD formation by macrophages also involves the activation of the signaling protein kinases PKC, PI3K, MEK1/2 and JNK, as well as the participation of phospholipases D (PLD) and induced iPLA_2_s. The authors also reported that CB induces the production of 15-d-PGJ_2_, a PGD_2_ precursor eicosanoid that presents an anti-inflammatory character via COX-1. Moreover, it was verified that CB-induced LDs compartmentalizes both COX-1 and 15-d-PGJ_2_, suggesting LDs as the site production of the pro-resolving mediator [85].

Although the above mentioned works associate CB with the production of eicosanoids (PGD_2_, 15-d-PGJ_2_ and PGE_2_) with possible anti-inflammatory activity [86–88], their biosynthesis were not associated with the immunomodulatory effects of CB. Otherwise, several others studies (already mentioned in this review) have endorsed that lipoxygenase-derived lipid mediators are key features in the immunomodulatory effects of both Crotoxin and CB. The lipoxygenase pathway is responsible for the generation of arachidonic acid-derived lipid mediators: leukotrienes, lipoxins and resolvins. These mediators may present ambiguous actions on inflammation, in which lipoxins and resolvins are associated with anti-inflammatory effects and are only increased during the resolution of inflammation [89]. Regarding the studies with Crotoxin and CB, the experimental procedures conducted in order to evaluate the participation of lipoxygenase-derived mediators consisted in applying pharmacological approaches that are capable of interfering on lipoxygenase biosynthesis and receptor binding, as well as the direct quantification of mediators.

During biosynthesis, the 5-, 12- and the 15-lipoxygenases are the major enzymes involved in the lipoxygenase-derived mediator production [90]. The drug nordihydroguaiaretic acid (NDGA) is a cell permeable phenolic agent that selectively inhibits lipoxygenase (LO) enzymes, with higher inhibitory efficiency to 5-LO. Nunes and colleagues [51] observed that NDGA reverted the decrease of peritoneal leukocyte migration induced by Crotoxin upon carrageenan stimulus. Another LO pathway inhibitor used was Zileuton, a selective 5-LO inhibitor that suppressed the inhibitory effects of Crotoxin and CB on macrophage phagocytosis (and the intracellular mechanisms involved in this event) and abolished the decrease in the number of circulating leukocytes and the increase of rolling leukocytes induced by Crotoxin and CB [55, 71].

Among lipoxygenase-induced mediators, LXA_4_ levels were increased in TNBS-treated colonic tissue of animals injected with Crotoxin as well as culture macrophages and dendritic cells treated with Crotoxin and CB [52, 55, 57]. LXA_4_ is a pro-resolving molecule secreted by immune cells such as neutrophils and macrophages, and 5- and 15-LO are important for its biosynthesis from arachidonic acid metabolism [90]. LXA_4_ dual anti-inflammatory and pro-resolving effects are mediated through binding to G protein-coupled LXA_4_ receptor (ALX) and formyl peptide receptor (FPR2). The lipoxygenase-induced mediator has been found to ameliorate leukocyte-mediated injuries by suppressing a wide range of leukocyte functions, such as chemotaxis, phagocytosis and the production of pro-inflammatory cytokines and cell proliferation [91–93].

Aside from using inhibitory drugs of the lipoxygenase biosynthesis pathway, authors have also applied the drug Boc2 (N-tert-butoxycarbonyl-FLFLF), a peptide mimetic antagonist of formyl peptide receptors (FPRs). The FPRs are G protein-coupled chemoattractant receptors with important roles in host defense and inflammation. Among the agonists, the lipoxygenase-derived mediator LXA_4_ presents high binding affinity to FPR2, however it does not activate the pro-inflammatory activities associated with this receptor, such as chemotaxis, enzyme release and ROS production. This intriguing aspect is considered the mechanism by which LXA_4_ acts as an anti-inflammatory agent when associated with FPR-interaction [94]. Nunes and colleagues [51] described that Boc2 reverted the decreased adherence and migration of leukocytes, and the increased leukocyte rolling induced by Crotoxin in animals stimulated with carrageenan. Moreover, Freitas and colleagues [57] demonstrated that Boc2 was able to regress the inflammatory resolution properties of Crotoxin on the dendritic cell maturation induced by LPS, characterized by the inhibition of MHC-II and costimulatory molecules CD40, CD80 and CD86, the decrease of the pro-inflammatory mediators IL-12, TNF-α and IL-6, as well as the increase of the anti-inflammatory mediators IL-10, PGE_2_ and LXA_4_, all induced by the toxin.

**Fig. 4:**
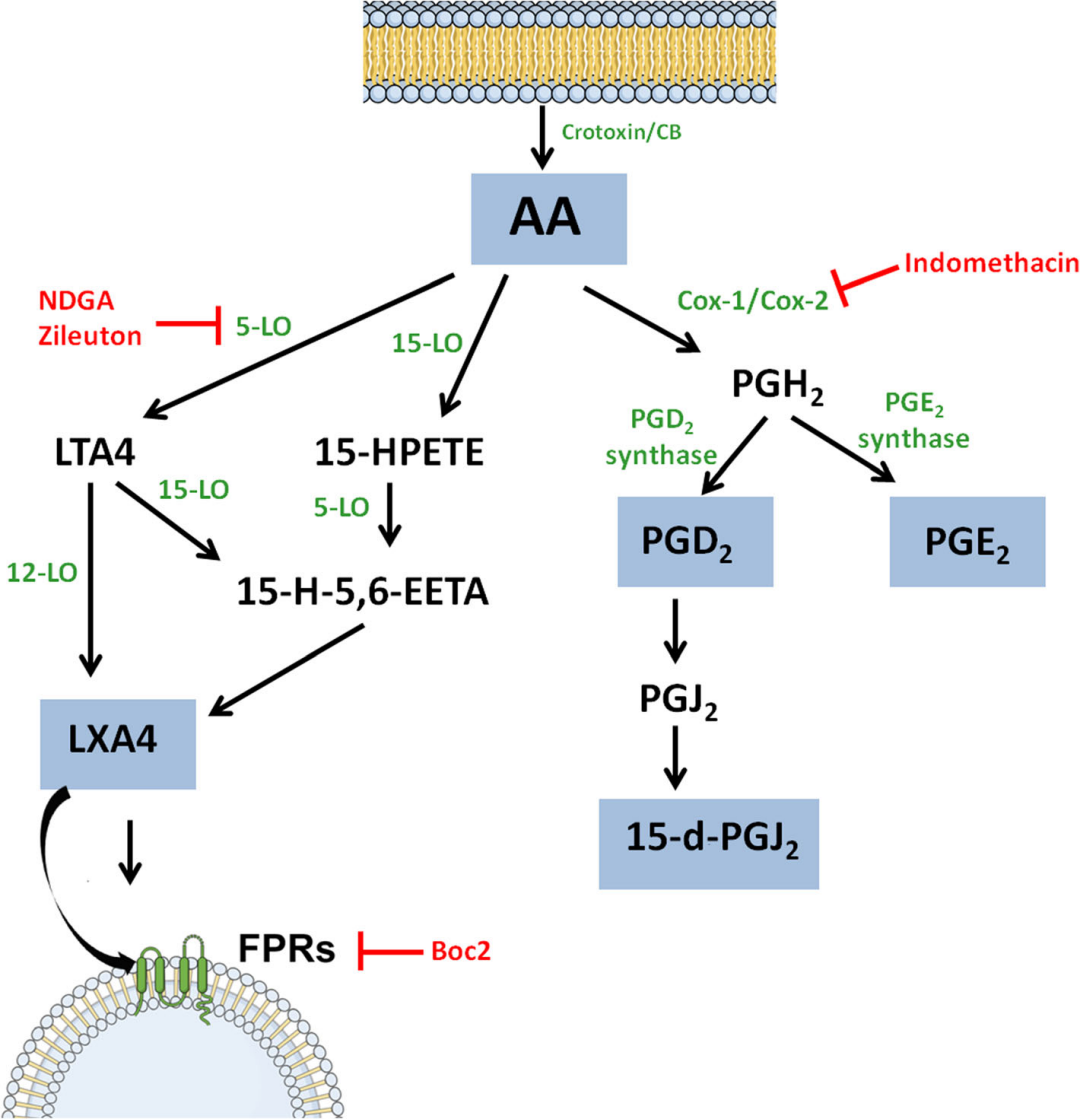
Eicosanoid biosynthesis. Eicosanoid biosynthesis from arachidonic acid induced by Crotoxin and its subunits and the turning points in which some pharmacological drugs act. Enzymes are represented in green, eicosanoids produced by Crotoxin or CB are shown within blue boxes and inhibitory drugs are represented in red. LTA_4_ (Leukotriene A_4_), 15-HPETE (15-Hydroxyeicosatetraenoic acid), 15-H-5,6-EETA (15-Hydroxy-5,6-epoxyeicosatrienoic acid)

Summing up, the results obtained from the reports described above clearly indicate that Crotoxin and its subunits are capable of inducing a leukocyte mediated biosynthesis of lipid mediators that exert immunomodulatory effects. The Lipoxin A_4_ is possibly the pivotal agent responsible for the anti-inflammatory and immunosuppressive activity, and is intrinsically associated with Crotoxin and CB phospholipase catalytic activity. A schematic representation of the biosynthesis and the turning points in which some pharmacological drugs act is illustrated in Fig. 4.

## Conclusion

In toxinology, the history of studying Crotoxin’s involvement in immunology shows the pursuit for better understanding the pathophysiological mechanisms of the major toxic component of *Crotalus durissus terrificus* venom and its association with envenomation and antivenom production. In addition, the capacity of Crotoxin and its subunits to modulate the immune system introduced novel perspectives as potential therapeutic agents to be applied on immune-associated diseases. Several studies have shown that these toxins modulate the cellular events of both innate and humoral immunity by producing endogenous lipid mediators with anti-inflammatory and immunosuppressive effects associated with pro-resolving mechanisms of immunological-related disturbs. Moreover, the potential immune effects of such toxins could be explored to ameliorate the conditions of other immune-associated disturbs, such as arthritis, diabetes, multiple sclerosis and behavioral disorders, opening novel perspectives for possible therapeutic applications. Nevertheless, further efforts are still necessary in order to better understand Crotoxin’s effects. Different possible mechanisms of action, such as the involvement of neuro-immune axis in its immunomodulatory effects, should also be investigated. As for the toxicological approach, important issues in the use of Crotoxin and its components should be standardized, such as administration routs and doses, as well as the investigation of possible biochemical and hematological alterations. All integrative approaches are important, aiming at a possible medicinal application of Crotoxin against human diseases.
